# Development of the Woman-Centred Care Scale- Midwife Self Report (WCCS-MSR)

**DOI:** 10.1186/s12884-021-03987-z

**Published:** 2021-07-23

**Authors:** Deborah L. Davis, Debra K. Creedy, Zoe Bradfield, Elizabeth Newnham, Marjorie Atchan, Lorna Davie, Judith McAra-Couper, Kristen Graham, Christine Griffiths, Linda Sweet, Virginia Stulz

**Affiliations:** 1 Trans-Tasman Midwifery Education Consortium, ACT Government Health Directorate and University of Canberra, Faculty of Health, 11 Kirinari St, Bruce, ACT 2617 Australia; 2 Trans-Tasman Midwifery Education Consortium, Griffith University, School of Nursing and Midwifery, University Drive, Meadowbrook, QLD 4331 Australia; 3 Trans-Tasman Midwifery Education Consortium, Curtin University and King Edward Memorial Hospital, School of Nursing, Midwifery and Paramedicine, Hayman Rd, Bentley, WA 6102 Australia; 4 Trans-Tasman Midwifery Education Consortium, Griffith University, School of Nursing and Midwifery, University Drive, Meadowbrook, QLD 4131 Australia; 5 Tasman Midwifery Education Consortium, University of Canberra, Faculty of Health, 11 Kirinari St, Bruce, ACT 2617 Australia; 6 Trans-Tasman Midwifery Education Consortium, Ara Institute of Canterbury Ltd, 276 Antigua St, Christchurch, 8140 New Zealand; 7 Midwifery Department, Trans-Tasman Midwifery Education Consortium, Auckland University of Technology, 640 Great South Road, Manukau, Auckland, 2025 New Zealand; 8 Trans-Tasman Midwifery Education Consortium, Flinders University, College of Nursing and Health Sciences, GPO Box 2100, Adelaide, SA 5001 Australia; 9 Trans-Tasman Midwifery Education Consortium, Otago Polytechnic, School of Midwifery, Forth Street, Dunedin, New Zealand; 10 Trans-Tasman Midwifery Education Consortium, Deakin University and Western Health Partnership, School of Nursing and Midwifery, School of Nursing and Midwifery, 221 Burwood Highway, , Burwood, Vic 3125 Australia; 11 Trans-Tasman Midwifery Education Consortium, Western Sydney University & Nepean Blue Mountains Local Health District, Court Building – Nepean Hospital, Centre for Nursing and Midwifery Research, Nepean Blue Mountains Local Health District, PO Box 63, Penrith, NSW 2751 Australia

**Keywords:** Woman-centred care; Pregnancy, Midwifery, Self-report, Surveys and tool, Instrument

## Abstract

**Background:**

Woman-centred care is recognised as a fundamental construct of midwifery practice yet to date, there has been no validated tool available to measure it. This study aims to develop and test a self-report tool to measure woman-centred care in midwives.

**Methods:**

A staged approach was used for tool development including deductive methods to generate items, testing content validity with a group of experts, and psychometrically testing the instrument with a sample drawn from the target audience. The draft 58 item tool was distributed in an online survey using professional networks in Australia and New Zealand. Testing included item analysis, principal components analysis with direct oblimin rotation and subscale analysis, and internal consistency reliability.

**Results:**

In total, 319 surveys were returned. Analysis revealed five factors explaining 47.6% of variance. Items were reduced to 40. Internal consistency (.92) was high but varied across factors. Factors reflected the extent to which a midwife meets the woman’s unique needs; balances the woman’s needs within the context of the maternity service; ensures midwifery philosophy underpins practice; uses evidence to inform collaborative practice; and works in partnership with the woman.

**Conclusion:**

The Woman-Centred Care Scale-Midwife Self Report is the first step in developing a valid and reliable tool to enable midwives to self-assess their woman-centredness. Further research in alternate populations and refinement is warranted.

## Introduction

Woman-centred care is a concept that emerged from the women’s health movement of the 1970s and 80 s underpinned by a feminist ethic [1]. It can be defined as care that “focuses on the woman’s unique needs, expectations and aspirations; recognises her right to self-determination in terms of choice, control and continuity of care; and addresses her social, emotional, physical, psychological, spiritual and cultural needs and expectations”[2]. This can be contrasted for example, with care that focuses on the needs of the health service or health professionals. In many countries, woman-centred care has become a defining feature of midwifery. This is reflected in the International Confederation of Midwives (ICM) (2014) philosophy of midwifery care. In Australia and New Zealand, woman-centred care in midwifery practice is widely recognised. Woman-centred care is embedded in regulation as part of the *Midwife Standards for Practice* in Australia [3] and *Competencies for Entry to the Register of Midwives* in New Zealand [4], education standards for the accreditation of midwifery programs [5, 6], as part of midwifery philosophy [7, 8], and in practice informing clinical care guidelines [9]. It is also central to Australia’s strategic direction for maternity services [10]. The fundamental nature of woman-centred care to midwifery practice is also espoused by peak professional midwifery organisations in countries such as the United Kingdom (UK) [11], Canada [12], and the United States of America (USA) [13].

While a body of research uses the concept of woman-centred care [14] there has been surprisingly little work on analysing or defining the concept, and less still on its measurement. Recent phenomenological research [15] revealed that woman-centred care is the practical manifestation of working within the broader midwifery philosophy of being ‘with woman’. In-depth interviews with 31 midwives working in a variety of models revealed numerous practice attributes of woman-centred care. Attributes included advocacy, advanced communication skills, facilitation of informed decision making, adaptability, creating a ‘space’, midwifery presence, preparing the environment, personal qualities, and therapeutic touch. Maputle and Donavon [16] also undertook a concept analysis, identifying the defining attributes of woman-centred care as: mutual participation and responsibility sharing, information sharing and empowering, communication and listening, accommodative midwifery actions, and maximising human and material infrastructure. An advanced concept analysis by Fontein-Kuipers, de Groot [17] highlighted the consciousness of choosing to work in a woman-centred way, the collaborative relationship between midwife and woman, mutual respect for each other’s knowledge, and equal focus on experience, meaning and manageability, and the health and wellbeing of mother and child.

A recent integrative review identified 17 studies that addressed woman-centred care as an intervention or outcome [18]. Sub-themes were identified under three pre-determined themes: clinical practice, models of care and education. From the theme of clinical practice, the sub-themes identified were choice and control, empowerment, protection of normal birth, relationship, and characteristics of the midwife. While there is variation in the components and definitions offered in these reviews and analyses, there is also a high degree of overlap, especially regarding the nature of the midwife-woman relationship, and empowerment of the childbearing woman. It is difficult to quantify the outcomes of woman-centred care for childbearing women, without an adequate instrument to measure the concept, though empowerment of the childbearing woman is a recurring theme [19–21].

Given the centrality of the concept of woman-centred care to midwifery, it is important that it can be measured. Measurement could determine, for example, the extent to which midwives are practicing woman-centred care, whether certain student experiences or curricula enhance woman-centred approaches, or whether models of care, employment modes, or practice environments impact woman-centred care. While several instruments measure patient or person-centred care [22, 23] and there is some over-lap, these are inadequate for capturing the uniqueness of midwifery practice and the feminist ethic that informs midwifery and the concept of woman-centred care. For example, one tool included items related to skill mix; how the health professional operates in the clinical environment; shared decision-making amongst the team (but not with the woman); effective staff relationships, power-sharing (rather than working in partnership) and availability of supportive organisational systems [19]. Brady, Bogossian [24] developed and pilot tested an instrument to measure woman-centred care behaviour in student midwives, mapping 18 behaviours across four core concepts: woman’s sphere, holism, self-determination, and shared power relationship. This instrument was tested within the context of a clinical simulation, with assessment of student performance completed by expert clinicians. This work makes an important contribution in the context of midwifery education, though the broader application is limited by the requirement for observation and assessment by a third party. The current study addresses this limitation, focusing on the development of a tool to measure midwives’ self-reported woman-centredness.

## Methods

A staged multi-method approach was used for tool development. In stage one, deductive methods were used for item generation, in stage two, content validity was established by a group of experts and in stage three, the instrument was completed by a sample drawn from the target audience and responses psychometrically tested.

### Stage 1- Item generation

Having a clear understanding of what is to be measured and generating an item pool are central processes in tool development [25]. Comprehensive reviews of the concept of woman-centred care have been undertaken [16–18]. Therefore, a deductive item generation process began with constructs identified in these reviews. A construct is defined here as a component of woman-centred care such as joint decision-making or reciprocity. From the literature, 25 [18], 85 [16] and 53 [17] constructs were identified including those duplicated within and between reviews.

The constructs identified from the literature were organised into domains. A domain is defined here as an overarching category in which similar constructs are grouped, according to a midwifery behavior or attitude that constitutes woman-centred care (as identified in the literature review) and items, as statements that address the constructs. Four domains and 19 constructs were identified. These are presented in Table 1.

**Table 1:** Domains and constructs

**Domain 1**	**Fosters a partnership relationship**
*The midwife is knowledgeable, skilled, and confident, which engenders trust and a sense of safety for the woman. The woman feels safe sharing information because the midwife protects her privacy. The midwife uses a variety of interpersonal skills to foster a partnership relationship characterised by reciprocity and equality. The midwife is nonjudgmental, respectful and shows empathy towards the woman.*	
**Constructs **	The midwife is knowledgeable, skilled, and confident; engendering trust and a sense of safety
	The midwife protects the woman's privacy
	The midwife fosters a partnership relationship based on honesty, openness, reciprocity, and equality
	The midwife is nonjudgmental, respectful and shows empathy towards the woman
**Domain 2**	**Provides care that is wellness focused**
*The midwife approaches childbearing through a lens of wellbeing, identifying and promoting salutary factors, optimising outcomes, and providing the woman with a positive experience. The midwife supports, protects, and enhances the physiological processes of childbearing, recognising the influence of environment on the woman's outcomes and experiences. The midwife recognises the health gains from building the woman's capacity and confidence in her own abilities.*	
**Constructs **	The midwife frames practice through the lens of wellbeing
	The midwife supports, protects, and enhances the physiological processes of pregnancy, birth and the postpartum
	The midwife recognises the influence of environment
	The midwife is focused on providing the woman with a positive experience
	The midwife has confidence in the woman's abilities fostering self-confidence in the woman
**Domain 3**	**Promotes woman's autonomy and empowerment**
*The midwife recognises a woman’s autonomy and right to self-determination. The midwife facilitates the woman’s engagement in decision making processes and provides unbiased information to promote informed choice and consent. The midwife upholds the woman’s central role in decision making, the requirement for fully informed consent and advocates for her when necessary.*	
**Constructs **	The midwife advocates for the woman when necessary
	The midwife recognises the woman’s autonomy and right to self determination
	The midwife provides unbiased information to facilitate informed choice and consent
	The midwife recognises the woman's central role in decision making
	The midwife facilitates engagement, informed choice and maternal control
**Domain 4**	**Provides care that is individualised and holistic**
*The midwife encourages the woman to articulate her needs and desires so that care can be tailored to her needs. The woman’s needs are prioritised. The midwife individualises information and communication strategies to promote understanding. Each woman is considered within the context of her social situation including the people important to her, family and community. The midwife approaches the woman holistically considering her emotional, social, psychological, and spiritual wellbeing as much as her physical.*	
**Constructs **	The midwife encourages the woman to articulate her ideas and needs
	The midwife provides care that is tailored and adaptive to the woman's needs
	The midwife puts the needs of the woman first
	The midwife respects the woman's culture and values
	The midwife responds to the woman holistically

It is recommended that researchers take a liberal approach to item generation with the item pool at least twice as long as the desired scale [26]. In total 98 items were generated. To minimise response bias, around 40% of items were negatively worded (e.g. “*Guidelines influence me to intervene even when I don’t think it is necessary*”). Two response scales were applied. A seven-point Likert scale assessed the extent to which an item reflected a respondent’s beliefs and practice of 1 = ‘very untrue of me” to 7 = “very true of me”. Other items had a response scale according to level of agreement from 1 = “strongly disagree” to 7 = “strongly agree”. In both cases, a seven-point response scale was chosen to foster meaningful discrimination along the response continuum on an underlying attribute/construct [25].

### Stage 2—External expert review: content validity

Content validity of draft items were conducted by an external expert panel. The research team distributed an invitation to midwives within their networks with known midwifery expertise. In total 14 experts responded with ten members from Australia and four from New Zealand. Panel members received a copy of the draft survey by email with instructions on how to complete and rate items.

Items were judged individually for clarity and relevance on a 4-point scale of: 1 = Strongly Disagree, 2 = Disagree, 3 = Agree, and 4 = Strongly Agree. Responses were uploaded into an Excel spreadsheet. A content validity index (CVI) was calculated based on the percentage of total items rated by experts as either 3 or 4. A CVI above 85% was considered valid [27]. The research team considered the feedback and ratings by the external expert panel and reduced the number of items from 98 to 58. The CVI analysis revealed 86–90% agreement on included items.

### Stage 3-Survey and psychometric testing

The survey was distributed to Australian and New Zealand midwives for the purpose of testing the tool.

#### Participants

Criteria for survey participation were midwives registered in Australia or New Zealand.

#### Sample size

A large sample is desirable for scale development. Sample size was calculated using (1) a ratio of at least five participants per variable (that is 5 × 58 items = 290) [28], and (2) specific statistical processes to ensure rigour. Setting high communalities with strong loadings (> 0.3) and limited cross loadings in factor analysis, ensure low error, and minimises small sample size bias [29].

#### Survey content

Part 1 of the survey asked about professional and personal characteristics. Details included: age; country of practice; hours of work; practice model; employment conditions; employment sector; primary area of practice (full scope, antenatal, labour and birth, postpartum, other); details of education; continuity of care (CoC) episodes during education; and perceived value of CoC experiences during education. Categories for each of the above can be found in Table 2. Part 2 of the survey presented the 58-item draft tool.

**Table 2:** Participant characteristics (n = 319)

Characteristic	Range/Mean (SD) n (%)
**Age**	22- 67/43.5 (11.36)
**Country of practice**	
Australia	280 (87.8)
New Zealand	39 (12.2)
**Years of practice**	
New graduate to 4 years	78 (24.4)
5 to 10 years	80 (25.0)
11 to 20 years	65 (20.4)
21 to 30 years	53 (16.6)
> 31 years	43 (13.5)
**Hours worked per week**	
Up to 16 h	40 (12.5)
17 to 32 h	94 (29.4)
33 to 40 h	125 (39.2)
> 40 h	60 (18.8)
**Practice model**	
Caseload/Continuity of Midwifery Care	104 (32.6)
Shift work	140 (43.9)
Other	75 (23.5)
**Main income**	
Self-employed	44 (13.8)
Permanent employed	239 (74.9)
Casual	24 (7.5)
Other	12 (3.8)
**Employment sector**	
Public sector	253 (79.3)
Private Sector	16 (5.0)
Other	6 (1.9)
Missing	44 (13.8)
**Education Institution for registration**	
Hospital training program	100 (31.3)
Polytechnic/ University	214 (67.1)
Other	5 (1.4)
**Qualification for registration**	
Certificate	75 (23.5)
Bachelor’s degree	141 (44.2)
Postgraduate diploma	77 (24.1)
Masters	9 (2.8)
Other	17 (5.3)
**Number of CoC during pre-registration education**	
None	77 (24.1)
1 to 14 women	93 (29.2)
15 to 34	119 (37.3)
35 or more	30 (9.5)
**Perceived value of CoC experiences**	
High value	162 (66.9)
Moderate value	59 (24.4)
Neutral	6 (2.5)
Low/No value	10 (4.1)
Unsure /Don’t remember	5 (2.1)

#### Procedure

Potential participants were contacted through professional networks of the research team and snowball sampling on Facebook. Willing participants were sent an email informing them about the research project, and its applicability to midwifery practice. The email included a link to the survey which was uploaded on the Qualtrics platform [30]. Undertaking the survey implied consent and participants could stop participating at any time. Participants were asked to record their first, instinctive answer and not think about what their answers “should” be.

#### Ethical considerations

Approval was granted by the University of Canberra Human Research Ethics Committee (Application number 2312). The survey was anonymous and no names or identifying information were required.

#### Approach to analysis

Data were cleaned and checked. Survey forms with missing values for any scale item were not included in the analysis. Some variables were recoded such as years of practice and hours worked per week to enable common categorisations and ease of description. Negatively worded items were reverse coded. Inspection of the correlation matrix was performed to assess feasibility for construct validity as factor analysis. Items with an average inter-item correlation of above 0.30 were considered valid [31]. In preparation for the principal components analysis (PCA), Kaiser–Meyer–Olkin (KMO) measure of sampling adequacy was deemed very good at 0.92 [32]. The Bartlett's Test of Significance was significant (5030.03, p < 0.001) enabling factor analysis [32]. Direct oblimin with Kaiser rotation was used to improve interpretability of the factors and further refine the groupings of items. Accordingly, all components with eigenvalues under 1.0 were dropped. All communalities were above the set cut-off value of 0.3. The criterion for factor extraction was an eigenvalue of > 1 and item factor loading of > 0.30 [25]. Correlations between factor and total scale scores as well as item-subscale correlations were calculated. Internal consistency for reliability was assessed using Cronbach’s alpha reliability coefficient where 0.6 was considered low, 0.7 acceptable and 0.8 or more as high. The final 40 items met these criteria. Descriptive statistics analysed characteristics of the sample and survey responses. Total scores were calculated for the scale and each subscale. Subscale scores were interpreted as low (less than 60% of possible total), moderate (60—80% or less of possible total), and high (81 -100% of possible total). Pearson’s correlation coefficients tested associations amongst continuous scores. Coefficient values between 0.50 and 1 are considered strong; 0.30 to 0.49 are considered moderate; and values ± 0.29 are small. Data were analysed using the Statistical Package for the Social Sciences (SPSS) 26.0 (2020) personal computer version.

## Findings

Four hundred respondents commenced the survey, and 319 completed all the scale items. A response rate was unable to be determined due to the use of professional networks and snowball sampling. The sample met the requirements of the sample size calculation. The mean age of participants was 43.5 years (SD 11.4), which is slightly younger than the mean age of midwives in Australia (46.9 years) [33] and New Zealand (47.8 years) [34] and the distribution was skewed to reflect the aging midwifery workforce. A greater proportion of respondents practised in Australia (87.8%) compared to New Zealand (12.2%). More than two-fifths (43.9%) did shift work, while a third (32.6%) worked in a caseload CoC model. Approximately 37.8% of midwives work in caseload CoC models [34] in New Zealand but signifies an over-representation of midwives working in these models in Australia. Nearly half held a Bachelor’s degree (44.2%) and almost a quarter possessed a post-graduate diploma (24.1%) which is consistent with data from New Zealand [34] but there is no available Australian data on this point. Participants also responded to “other” forms of main employment as full time or part time employment or contract work, graduate employment, agency work, being an educator and part owner of a business. Participant characteristics are presented in Table 2.

### Factor analysis

Principal component analysis extracted five factors with eigenvalues exceeding a value of one [32]. The first factor explained 29% of variance, the second, 5.9%, the third, 4.9%, the fourth, 4.2% and the fifth, 3.6% (47.6% total variance). Eigenvalues, communalities and retained items are displayed in Table 3.

**Table 3:** Factor analysis: pattern matrix, communalities, scale and factor reliability and means

Factor and item	Communality	Eigen-value	Cronbach’s Alpha	Mean (SD)
**Total scale** (40 items)			0.92	237.93 (38.78)
**Factor 1 – Meets the unique needs of the woman (12 items)**		11.60	0.89	73.89 (10)
73. I employ a variety of strategies to adapt the environment to meet a woman’s needs	0.59			6.17 (.79)
77. I put as much effort into creating a positive experience as I do in achieving a good clinical outcome	0.50			6.32 (.86)
75. I have strategies for creating safe spaces for the woman	0.41			6.11 (.93)
70. I always make suggestions to enhance the normal physiology of pregnancy, birth or postpartum	0.52			6.32 (.85)
68. No matter what the situation I can always find a way to enhance the woman’s wellbeing	0.54			5.73 (.91)
76. I pay attention to the impact of the physical environment on a woman’s dignity	0.52			6.51 (.62)
66. I am always comfortable discussing spirituality with women	0.42			5.57 (1.2)
63. I always discuss a woman’s cultural needs with her	0.57			6.10 (.88)
58. I am flexible in accommodating a woman’s wishes & needs	0.55			6.18 (.69)
65. I always establish who is important to the woman be that partner, family member or friend	0.51			6.33 (.76)
59. I always identify the woman’s values and include these in discussions about care options	0.55			6.15 (.75)
71. I always provide information, so the woman understands the physiological processes of her own body	0.51			6.40 (.70)
**Factor 2 – Balances the woman’s needs within the context of the maternity service (5 items)**		2.37	0.68	26.6 (7.56)
78. ^a^Women’s expectations for positive childbearing experiences are too high	0.39			5.63 (1.57)
26. I don’t always follow hospital policy or prevailing clinical guidelines if that is not what the woman wants	0.62			4.64 (1.88)
55. ^a^Standardising care with protocols ensures optimal outcomes for all women	0.53			4.55 (1.75)
57. ^a^The needs of the organisation mostly should take priority over the needs of the woman	0.45			6.28 (1.06)
29. The woman’s informed choice is more important than my professional expertise in making decisions	0.35			5.50 (1.28)
**Factor 3 – Ensures midwifery philosophy underpins practice within the context of the maternity service** **(4 items)**		1.967	0.63	17.43 (6.69)
72. ^a^Guidelines influence me to intervene even when I don’t think it is necessary	0.39			3.80 (1.60)
56. ^a^I can't always personalise the woman's experience	0.58			4.46 (1.62)
33. ^a^I struggle to maintain competence in all areas of midwifery practice	0.36			5.05 (1.80)
51.^a^ I am not always able to tailor care to the needs of each woman	0.65			4.12 (1.66)
**Factor 4 –Working collaboratively for evidence-based practice** **(7 items)**		1.66	0.73	41.65 (7.03)
35. I always recognise when a woman's care requires referral to another health professional	0.37			6.29 (.69)
37. I actively seek feedback from women/others to evaluate my practice	0.55			5.63 (1.33)
34. I always provide my professional opinion even if other health care professionals don’t agree	0.49			5.20 (1.27)
23. I regularly examine the potential for my personal bias or agenda to influence the informed choice process	0.42			5.79 (1.18)
36. I know where to find evidence-based information to inform my practice	0.42			6.27 (.85)
38. I challenge others when a woman’s privacy is being compromised	0.42			6.23 (.83)
25. Every clinical decision I make is in collaboration with the woman	0.42			6.24 (.87)
**Factor 5 – Works in partnership with the woman (12 items)**		1.44	0.84	77.61 (7.91)
27. I always document the woman's choices	0.33			6.56 (.60)
52. I always use language that matches the woman’s levesl understanding	0.44			6.22 (.67)
62. I am comfortable involving family members or significant others when a woman wants this	0.41			6.52 (.69)
43. Sharing of responsibility requires adequate information	0.35			6.57 (.61)
39. I seek to minimise power imbalances through my speech and body language	0.37			6.31 (.80)
54. I accept that a woman can change her mind at any time regarding care	0.44			6.64 (.50)
40. Partnership means working closely with the woman and her significant others	0.40			6.65 (.63)
49. I am guided by the woman in relation to the involvement of support people	0.46			6.72 (.49)
48. I am interested in what a woman tells me about what is important to her	0.62			6.73 (.49)
45. I treat all women with courtesy no matter their life circumstances or decisions	0.64			6.73 (.49)
44. The relationship between a midwife and woman can influence clinical outcomes	0.37			6.51 (.67)
50. I ask questions about the woman’s social situation so I can offer better support	0.42			6.32 (.73)

^a^Reverse scored items

Direct oblimin with Kaiser rotation was used to improve interpretability of the five factors and further refine the groupings of items (see the pattern matrix as displayed in Table 4).

**Table 4:** Component correlation matrix

Component	Factor 1	Factor 2	Factor 3	Factor 4	Factor 5
Factor 1	1.000	.135	.247	.336	.509
Factor 2	.135	1.000	.022	.077	.158
Factor 3	.247	.022	1.000	.152	.124
Factor 4	.336	.077	.152	1.000	.242
Factor 5	.509	.158	.124	.242	1.000

### Correlations between factors

Correlations between factor and total scale scores, as well as item-subscale correlations were calculated and show that all items were not independent of each other and are interrelated (see Table 4). Correlations between factors revealed the importance of “*Meets the unique needs of the woman*” (Factor 1) and other aspects of woman-centred care, especially “*Works in Partnership with the woman*” (Factor 5) (r = 0.51) and “*Working collaboratively for evidence-based practice*” (Factor 4) (r = 0.33). There was a weaker relationship with Factors 2 (Balances the woman’s needs within the context of the maternity service) and 3 (Ensures midwifery philosophy underpins practice within the context of the maternity service) both of which reflect the practices of midwives within the broader maternity care system.

According to Cronbach’s alpha levels, internal consistency for the scales varied from low (0.6 for two scales) to acceptable (> 0.7 for three scales)(as shown in Table 3) [35]. Overall reliability was 0.92. Descriptive statistics were calculated for the overall tool and for each individual subscale (see Table 3). Mean scores represent a composite score for all individuals on that particular factor and these summed scores preserve variation in the original data [36]. The tool was named the Woman-Centred Care Scale- Midwife Self Report (WCCS-MSR).

## Discussion

Development and preliminary testing of the WCCS-MSR is an important undertaking to describe and measure critical aspects of woman-centred midwifery practice. Woman-centred care underpins midwifery philosophy and is an integral component of the midwifery landscape in Australia, New Zealand, and many other countries. Both midwives and women acknowledge the importance of woman-centred care, and the trusting relationship established between the midwife, the woman, and her significant others [37]. In the current study, a relatively large sample of midwives participated, giving confidence to the generalisability of results. Compared to workforce data in Australia and New Zealand, [33, 34], participating midwives were similar for age and education, although over- represented by Australian midwives working in a caseload model of care.

Factor 1 “*Meets the woman’s unique needs*” consists of 12 items with good internal consistency. The mean score of 73.9 out of a possible 84 reflects a high level of agreement. These items reflect a midwife’s ability to adapt to the environment to meet a woman’s needs (items 73 and 75), promote her dignity (item 76) and enhance her wellness (items 68 and 70). Fahy, Foureur [38] established the midwifery theory “Birth Territory”, suggesting that when midwives use midwifery guardianship, they create the ideal territory for women to birth, that enhances their normal physiology, enables them to feel satisfied and to transition seamlessly into the postnatal period. Creating a safe environment for the woman is crucial to enhance informed decision-making so that she feels empowered and supported [15, 16]. Providing a respectful, calm and safe birthing space for the woman to welcome her newborn into a safe birth space has been found to enhance the bonding relationship between the woman and her baby [39]. This factor also considered the importance of inclusion of the woman’s significant others (item 65) as working in partnership with the woman that means being inclusive of her partner and support people [37].

Factor 2 “*Balances the woman’s needs within the context of the maternity service*” speaks to the inherent tensions experienced by midwives as they advocate for the needs of women when working in the maternity system. The mean score of 26.6 out of a possible 35 reflects a moderate level of agreement. This factor may need further testing due to its moderate to low internal consistency. In general, this factor focuses on meeting the individual needs of women within the context of a maternity service that is driven by a standardised approach (items 26 and 55 for example) which is often risk-adverse. Walsh [40] refers to standard maternity care as an “assembly line” shaped by industrial models. This tension between standardisation and individualisation has also been recognised by health sociologists [41] and at the centre of calls for an approach to humanise childbirth [42]. This tension may relate to dilemmas in loyalty and professional socialisation, contributing to friction between the woman, the midwife, and other practitioners. The low internal consistency of this factor may reflect diversity in respondents’ educational qualifications and commitment life-long learning, education, reflection, and self-awareness though this was not examined in this study. Low internal consistency may be a reflection of the small number of items; low interrelatedness between test items, or the possibility that the items are measuring more than one latent variable. The relatively lower mean score demonstrated for items in this factor may also reflect the challenges of providing woman centred care within organisational contexts.

Factor 3 refers to “*Ensuring midwifery philosophy underpins practice within the context of the maternity service*” and has 4 items which were all worded negatively. The mean score of 17.4 out of a possible 28 reflects a modest level of agreement. This factor may also need to be reconsidered for further testing due to low internal consistency. The factor includes items focusing on a personalised approach to care (items 51 and 56), midwifery competence (item 33) and the influence of clinical guidelines (item 72). A personalised approach has been recognised by midwives as an important element of woman-centred care [15] although midwives must balance sometimes competing interests including organisational and interprofessional issues [43]. Another study also showed that midwives’ views have been strongly interwoven with advocacy for the woman, to ensure her choices are being met and promoting self-determination in order to support the woman’s accomplishment of her goals and choices, irrespective of the care setting [44]. Maintaining competence across all areas of midwifery practice may promote a personalised approach within these competing interests [45]. Like factor 2, the relatively lower mean score demonstrated for items in this factor may also reflect the challenges of providing woman centred care within organisational contexts.

Factor 4 “*Working collaboratively for evidence-based practice*” describes the collaborative nature of midwives’ work and the importance of evidence-based practice (EBP). The mean score of 41.6 out of a possible 49 reflects a high level of agreement. The seven items describe woman-centred midwifery practice as making decisions in collaboration with women (item 25); seeking feedback from women (item 37); and providing a professional opinion even if other health care professionals don’t agree (item 34). Woman-centred care is ascribed to mutual responsibility and participation that is demonstrated by interdependent collaboration, consultation and co-operation between the woman and the midwife [16]. Midwives as advocates for the women in their care, often face opposition from other health professionals including obstetricians and other midwives [43]. This factor seems to speak to confidence as knowing where to find evidence to inform practice, understanding professional boundaries, proffering one’s own professional opinion and challenging others, are characteristics of a confident midwife. Midwifery confidence has been linked to autonomy with workplace culture and midwifery colleagues being some of the most influential factors [45].

Factor 5 refers to “*Working in Partnership with Women*”. The mean score of 77.6 out of a possible 84 reflects a high level of agreement. This 12-item scale includes items related to informed choice and participation in health care decisions (items 27 and 52), inclusion of the woman’s significant others (items 62 and 49), and an approach to care that considers the woman holistically (items 48 and 50). The importance of respecting women’s autonomy in childbirth has long been understood and includes encouraging participation in health care decisions which is central to the partnership model of midwifery [46]. Involvement in decision making is associated with a greater sense of safety in childbirth and the World Health Organization (2016) has identified respect and autonomy as key features of quality maternity care.

Reliable and valid measurement of woman-centred care has several applications. The WCCS-MSR will be useful in contemporary practice to not only highlight best practice by midwives but identify areas in need of improvement. Repeating the tool over time could be included in professional development and as a reliable indicator of quality care. The WCCS-MSR could be used in pre-registration midwifery programs to make the elements of woman-centred care explicit to students, enable self-assessment, as well as identify areas for improvement. Woman-centred care is a universal principle and as such the WCCS-SR has applicability to all English-speaking midwives in any maternity care context.

### Limitations

There are several limitations associated with this preliminary study. Although a relatively large sample was recruited, 81 respondents (20%) commenced but did not complete the survey introducing possible response bias. Consequently, respondents may systematically differ from those who withdrew or did not respond. Additionally, the number of midwives invited to participate through professional networks and Facebook could not be determined, therefore a true response rate could not be calculated. The inability to contact respondents also precluded test–retest reliability being assessed. Generalisability may be limited as this survey was only distributed in Australia and New Zealand. The recruited sample was over-represented by Australian midwives working in a caseload model of care. The over representation may reflect the interest of caseload midwives in woman-centred care, introducing possible bias. Although the tool development team were geographically diverse, the overall network may have been homogenous thereby limiting the reach to different sectors of the midwifery community. Concurrent validity could not be established as the research team were unable to locate a similar tool in the literature. To this point, the topic has been primarily investigated through qualitative studies.

### Future directions

This is the first tool of its kind to enable midwives to undertake self-appraisal of their woman-centred practices. It is envisaged that the tool will be useful for reflective practice by midwives as they consider ways to develop skills and ways of caring that are aligned with professional philosophy and standards.

The team undertook rigorous tool development processes involving an analysis of systematic review findings; generation of a large pool of items; consultation with a group of experts; and piloting with a large sample of practicing midwives. WCCS-MSR items loaded on a five-factor solution which accounted for over 48% of the variance. While this proportion is acceptable, future research should aim to increase the variance accounted for, by refining existing items and adding new items where necessary. Various forms of validity testing reduced the number of items from 98 to 40 but other items that accurately reflect woman-centred midwifery care need to be included and tested. Two factors emerged with low internal consistency and further consideration of these items and testing is required. This may be achieved by validating this instrument with a larger and more diverse sample.

In this study, elements of validity were established (face, content, and construct) but further testing is required. The next step would be to test the WCCS-SR with another large diverse sample of midwives and include standardized tools that aim to measure similar (concurrent validity); different (discriminatory validity); or other constructs that predict woman-centred care. The total mean WCCS-MSR score of 237.9 out of a possible 280 reflects a high level of agreement on items. Future research could examine the internal structure of the WCCS-MSR using Rasch Measurement Theory [47, 48]. This analysis could inspect the response format, item fit, and differential item functioning to further validate the tool. Future distribution to other countries outside Australia and New Zealand is recommended.

## Conclusions

Woman-centred care is central to midwifery and it is timely that an instrument is developed to measure the behaviours that provide it. This study represents the first steps in this process. The five-factor solution contains items that resonate with descriptions of midwifery practice in the literature. The mean score achieved on the scale as a whole, reflects a high level of agreement though two factors demonstrated low or low to moderate internal consistency. Further work is required to refine and develop the WCCS-MSR.

## Data Availability

The datasets generated and analysed during the current study are not publicly available due to the parameters of ethical approval granted but may be available from the corresponding author on reasonable request.

## Abbreviations

CVI: Content validity index
CoC: Continuity of care
EBP: Evidence-based practice
ICM: International Confederation of Midwives
KMO: Kaiser-Meyer-Olkin
WCCS-MSR: Woman-Centred Care Scale- Midwife Self Report
PCA: Principal components analysis
UK: United Kingdom
USA: Unites States of America
